# Muscle IGF-1-Induced Skeletal Muscle Hypertrophy Evokes Higher Insulin Sensitivity and Carbohydrate Use as Preferential Energy Substrate

**DOI:** 10.1155/2015/282984

**Published:** 2015-02-04

**Authors:** Marcelo Augusto Christoffolete, William Jose Silva, Gracielle Vieira Ramos, Mirella Ribeiro Bento, Monique Oliveira Costa, Miriam Oliveira Ribeiro, Maristela Mitiko Okamoto, Tania Helena Lohmann, Ubiratan Fabres Machado, Antonio Musarò, Anselmo Sigari Moriscot

**Affiliations:** ^1^Department of Anatomy, Institute of Biomedical Sciences, University of São Paulo, 05508-000 São Paulo, SP, Brazil; ^2^Centro de Ciências Naturais e Humanas (CCNH), Universidade Federal do ABC (UFABC), 09210-580 Santo André, SP, Brazil; ^3^Biological and Health Science Center, Mackenzie Presbyterian University, 01302-907 São Paulo, SP, Brazil; ^4^Department of Physiology and Biophysics, Institute of Biomedical Sciences, University of São Paulo, 05508-000 São Paulo, SP, Brazil; ^5^Institute Pasteur Cenci Bolognetti, DAHFMO, Unit of Histology and Medical Embryology, IIM, Sapienza University of Rome, 00161 Rome, Italy

## Abstract

We characterized the metabolic profile of transgenic mice exhibiting enhanced muscle mass driven by increased mIGF-1 expression (MLC/mIGF-1). As expected, 6-month-old MLC/mIGF-1 mice were heavier than age-matched wild type (WT) mice (37.4 ± 0.3 versus 31.8 ± 0.6 g, resp.). MLC/mIGF-1 mice had higher respiratory quotient when compared to WT (0.9 ± 0.03 versus 0.74 ± 0.02, resp.) suggesting a preference for carbohydrate as the major fuel source. MLC/mIGF-1 mice had a higher rate of glucose disposal when compared to WT (3.25 ± 0.14 versus 2.39 ± 0.03%/min, resp.). The higher disposal rate correlated to ∼2-fold higher GLUT4 content in the extensor digitorum longus (EDL) muscle. Analysis of mRNA content for the glycolysis-related gene PFK-1 showed ∼3-fold upregulation in MLC/mIGF-1 animals. We also found a 50% downregulation of PGC1*α* mRNA levels in MLC/mIGF-1 mouse EDL muscle, suggesting less abundant mitochondria in this tissue. We found no difference in the expression of PPAR*α* and PPAR*β*/*δ*, suggesting no modulation of key elements in oxidative metabolism. These data together suggest a shift in metabolism towards higher carbohydrate utilization, and that could explain the increased insulin sensitivity of hypertrophied skeletal muscle in MLC/mIGF-1 mice.

## 1. Introduction

One of the remarkable features of skeletal muscle is the capacity to adapt its metabolic and functional properties in response to a wide range of external factors, including physical and neuron activity, change in hormone levels, and oxygen and nutrient supply [1]. However in several pathological conditions, skeletal muscle severely decreases this adaptive capacity, triggering alterations in its metabolic properties evolving to disease. In fact, insulin resistance (IR), obesity, high blood pressure, high fasting glucose or hyperglycemia, and lipid abnormalities are all conditions related to morphofunctional and metabolic changes in skeletal muscle [2].

Recent evidences have shown that different therapeutic interventions can improve body composition and systemic metabolism [3]. Among these, physical activity has been considered in the therapeutic effects of exercise for type II diabetes (T2DM) and other metabolic syndromes [4], although regular physical activity as a therapeutic tool can be seriously assumed in only a fraction of the population, mainly due to motivational and physical limitations. From a metabolic point of view, however, it remains not well understood what molecular/cellular aspects of exercise should be mimicked and what type of muscle fiber is best targeted to improve metabolic dysfunction. Increased physical activity is usually achieved by exercise training, which can be divided into two major categories: endurance and resistance. It is well known that endurance training promotes metabolic alterations in skeletal muscle due to a shift in substrate preference as a consequence of fatty acid oxidative metabolism and muscle fiber type interconversion, resulting in a greater number of the slow twitch oxidative fibers [5]. In contrast, resistance training is known for its capacity to promote an increase in skeletal muscle mass (hypertrophy) and strength [6]. Nonetheless, the molecular factors linking skeletal muscle growth and metabolism still remain to be explored in detail. Among growth factors, the Insulin-like growth factor-1 (IGF-1) has been implicated in the control of both muscle mass and skeletal muscle homeostasis and its expression is significantly enhanced in response to exercise [7].

It has been previously reported that muscle restricted mIGF-1 transgene (MLC/mIGF-1) sustains muscle hypertrophy and regeneration in senescent skeletal muscle, enhances the recruitment of circulating stem cells in injured muscle [8], and counteracts muscle wasting in mdx dystrophic mice [9], improving muscle mass and strength and elevating pathways associated with muscle survival and regeneration. Although significant advances have been made with this genetic model, overall, the impact of manipulating skeletal muscle hypertrophic pathways upon whole body metabolism and glucose disposal is largely unknown.

In the present study, we investigated metabolic features of MLC/mIGF-1 mice, revealing an unexpected higher respiratory quotient at rest, an index of higher carbohydrate utilization. These animals also showed higher insulin sensitivity, in line with a preference for carbohydrate as the primary energy source. In addition we have detected increased GLUT4 protein levels in skeletal muscle. These findings reinforce the potential of manipulating IGF-1 triggered intracellular pathways as therapeutic tools in the treatment of insulin resistance and type II diabetes.

## 2. Research Design and Methods

### 2.1. Animals

All procedures described were performed in accordance with the guidelines of the committee on animal research at the Institute of Biomedical Sciences, University of São Paulo. Animals aged 4–6 months were obtained from our colony at the Department of Anatomy animal facility. Mice were housed at 21°C on a 12 h dark/light cycle (lights on 18:00 to 06:00 h) with free access to food and water. At the end of the experiments, animals were anesthetized with a ketamine/xylazine combination (100 mg/kg and 10 mg/kg b.w., resp.), tissues were harvested and euthanasia performed by exsanguination cardiac arrest. Our MLC/mIGF-1 mice inbred colony was expanded from two heterozygote males donated by Dr. Musarò [8].

### 2.2. Genotyping

Approximately 0.5 cm of mouse tail tip was excised and submitted to total DNA extraction according to a protocol previously described [10], with the following modifications; tail biopsies were digested overnight at 55°C with agitation in lysis buffer (100 mM Tris-HCl pH 8.5, 10 mM EDTA pH 8.0, 0.5% SDS, and 200 mM NaCl) supplemented with 10 *μ*g Proteinase K (Invitrogen, Carlsbad, CA, USA). The resulting material was precipitated with isopropanol, washed in 70% ethanolandre-suspended in TE pH 8.0. Eighty nanograms of total genomic DNA was then submitted to conventional PCR. The reaction was carried out using a common sense primer targeting both mouse and rat MLC promoters (5′-GTGTCAAGGTTCTATTAGGCACTA-3′), and the presence of the transgenic gene was detected using a specific antisense primer targeting the mIGF-1 construct (5′-GAGCTGACTTTGTAGGCTTCA-3′); a positive control reaction was performed using a specific primer (5′-TGACCAAAACGATTCACCTG-3′) targeting the mouse MLC gene. Cycling conditions were 3 min at 95°C “hot start”, followed by 35 cycles at 95°C for 25 s, 60°C for 30 s, and 72°C for 60 s. The control amplicon consisted of ~300 bp fragment and the presence of the transgene was confirmed by identification of ~600 bp fragment, as determined by electrophoretic analysis.

### 2.3. Muscle Mass Determination, Histochemical Analysis, and Measurement of Cross-Sectional Area

At the time of animal sacrifice, soleus and extensor digitorum longus (EDL) muscles were carefully dissected from both limbs. The muscle was weighted and cut in half; one segment was immersed in cold isopentane for 30 seconds, cooled in liquid nitrogen, and stored at −80°C for histochemistry; the other segment was snap frozen in liquid nitrogen and stored at −80°C for later analysis. EDL and soleus frozen muscles were cut into 10 *μ*m cross-sections on a cryostat (CM3050; Leica, Nussloch, Germany) in order to determine the CSA of different fiber types by myofibrillar ATPase activity after alkaline (ATPase, pH 10.3) or acid preincubation (ATPase, pH 4.1) [11, 12]. The CSA (cross-sectional area) and relative total composition of each fiber type were obtained by measuring the CSAs of approximately 500 muscle fibers of each mouse (expressed as mean ± SEM) through image software Image Pro-Plus. The acquisition of the images was realized using a microscope (Nikon Eclipse TS100, Japan) equipped with a digital video camera and imaging software (NIS-Elements BR).

### 2.4. Fasting Blood Cholesterol, Triglycerides and Glucose Concentrations, and Carcass Lipid Content

Animals were fasted for 8 h, with food being removed at 08:00 h and the test begun at 16:00 h. Cholesterol and triglycerides were determined in spectrophotometer by colorimetric method using a commercial KIT (Labtest Diagnóstica S/A, Lagoa Santa, MG, Brazil). Glucose was determined using specific test strips in an Accu-Chek meter (Roche Diagnostics GmbH, Mannheim, Germany). For carcass analysis, lipid extraction was performed as described [13] and body fat percentage was determined.

### 2.5. Oxygen Consumption

Resting metabolic rate was estimated by determining oxygen consumption (VO_2_) in an open circuit respirometer (O2-10, Sable System, Las Vegas, NV, USA) [14, 15]. The measurements were conducted always in the afternoon (14:00–18:00 h) for 30 min at 25°C in animals fed* ad libitum*. The data were collected and evaluated by using the Sable Systems software. The results were expressed as milliliters of O_2_/min/grams of BW [16, 17].

### 2.6. Insulin Tolerance Test (ITT) and kITT Determination

For ITT, animals were deprived of food for 2 h. Standard insulin diluted in saline was injected (0.125 U/kg) via the caudal vein. Blood glucose was measured with a glucometer (Accu-chek, Roche) at 0, 6, 9, 12, and 15 minutes after insulin injection. The constant rate for blood glucose disappearance (kITT) was calculated based on the linear regression of the Napierian logarithm of blood glucose concentrations [18].

### 2.7. Real-Time Quantitative Polymerase Chain Reaction

Real-time quantitative polymerase chain reaction (PCR) was performed on an ABI Prism 5700 sequence detection system (Applied Biosystems, Foster City, CA, USA) using SYBR Green (Applied Biosystems) following the instructions provided by the manufacturer. Thermal-cycling conditions for the real-time PCR were 50°C for 2 min, 95°C for 10 min, and then 40 cycles of 95°C for 15 s, 60°C for 25 s, and 72°C for 30 s. The internal control, Cyclophilin A (CycloA), was amplified in separate tubes. The data were collected semiquantitatively together with the cycle threshold reading of corresponding CycloA internal controls. Data from five to six determinations (mean ± SEM) are expressed in all experiments as fold changes relative to the control group which was arbitrarily set as one. Primers are available upon request.

### 2.8. Western-Blot Analysis

Protein extracts from tissue samples were obtained by ultrasonic membrane disruption in lysis buffer, containing Tris-HCl (pH 7.5) 100 mM, EDTA 10 mM, SDS 1%, NaF 100 mM, Na_4_PO_2_O_7_ 10 mM, and sodium orthovanadate 10 mM, supplemented with protease inhibitor (Sigma). Protein concentration was determined by the Bradford method [19]. Fifty micrograms of total protein was loaded on a 12% SDS:acrylamide gel (SDS-PAGE) and separated by electrophoresis. Proteins were transferred to a nitrocellulose membrane in a semidry system and probed using a specific antibody for GLUT4 (1 : 1000; cat#2213; Cell Signaling) or GAPDH (1 : 1000; cat#2118; Cell Signaling) with incubation overnight at 4°C. Membrane was washed 15 min in TBS-T (Tween Tris-buffered saline solution) and incubated with secondary antibody goat anti-rabbit IgG (alkaline-phosphatase conjugated 1 : 1000; cat#D0487; Dako^©^, Glostrup, Denmark) for 1 hour at room temperature and posteriorly washed again for 15 min in TBS-T. Specific bands were visualized by enzymatic colorimetric NBT/BCIP (Roche, Mannheim, Germany).

### 2.9. Data Analysis

All data obtained were analyzed using GraphPad Prism version 5.00.288 (GraphPad Software Inc. San Diego, CA, USA) and plotted as graphs. Data were submitted to Student's *t*-test to compare WT and MLC/mIGF-1 animals. All data are expressed as mean ± SEM.

## 3. Results

**Figure 1:**
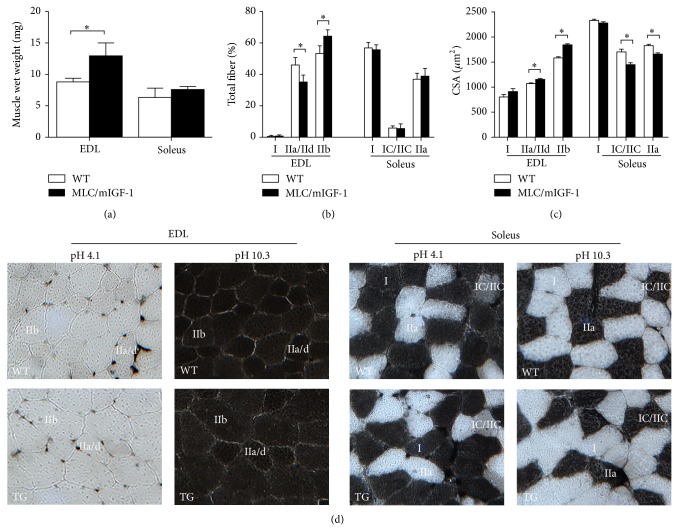
(a) Wet weight of EDL and soleus muscles from WT (open bars) and MLC/mIGF-1 mice (filled bars). (b) Composition of each fiber type in EDL (type I, IIa/IId and IIb) and soleus (type I, IC/IIC and IIa) muscles from WT (open bars) and MLC/mIGF-1 mice (filled bars). (c) Cross-sectional area-CSA (*μ*m^2^) of each fiber type in EDL and soleus muscles from WT (open bars) and MLC/mIGF-1 (filled bars) mice. (d) Serial cross-sections of the EDL and soleus muscles from WT and MLC/mIGF-1 mice muscle were submitted to histochemistry mATPase at pH 4.1 and 10.3. Data expressed as mean ± SEM. *n* = 4-5; ^*^ *P* < 0.05 versus WT muscles.

As expected, MLC/mIGF-1 mice [8] presented a very selective and marked hypertrophy in the EDL muscle, which predominantly contains type II fast twitch fibers. This is supported by the 1.47-fold increment in wet weight in MLC/mIGF-1 EDL muscle (Figure 1(a)); on the other hand, no change in soleus wet weight was observed. Histochemical analysis clearly demonstrated an increase in incidence of fiber type IIb and decrease in the incidence of fiber type IIa/IId in the EDL of MLC/mIGF-1 animals when compared to WT animals (Figure 1(b)); on the other hand, the predominance of fiber types in the soleus muscle did not change (Figure 1(b)). We found that CSA of fiber types IIa/IId and IIb are increased in EDL muscle of MLC/mIGF-1 (Figures 1(c) and 1(d)) mice. Curiously, CSA of type IC/IIC and IIa fibers decreased in soleus muscle as compared to WT animals (Figures 1(c) and 1(d)). Because we have found increases in wet weight and CSA only in the EDL, we decided to proceed with further measurements exclusively in this muscle.

Analysis of biometric parameters of MLC/mIGF-1 mice revealed a very significant increase in body weight (37.4 ± 0.3 versus 31.8 ± 0.6 g, *P* < 0.0001, MLC/mIGF-1 and WT, resp., Table 1). This appears to be a major consequence of skeletal muscle hypertrophy, since there were no differences in fat mass and body fat composition (Table 1). Also, although MLC/mIGF-1 animals were heavier we did not find a difference in length between the two groups (Table 1). It was intuitive to assume that MLC/mIGF-1 mice would sustain greater skeletal muscle mass at the expense of higher food consumption. Nevertheless, we observed the same food intake per animal (not shown) in both groups and, more interestingly, lower food consumption in MLC/mIGF-1 mice compared to WT when corrected for body weight (Table 1). Because these data suggested increased metabolic efficiency in the MLC/mIGF-1 model, we decided to test this hypothesis by indirect calorimetry. Our next series of experiments showed a trend towards lower O_2_ consumption in MLC/mIGF-1 animals (Table 1). Although not significantly different, this trend was accompanied by a significantly higher respiratory quotient (RQ) (0.74 ± 0.02 versus 0.90 ± 0.03; *P* < 0.001 WT and MLC/mIGF-1 animals, resp.) (Table 1). According to the literature, these data strongly suggest higher oxidation of carbohydrates [16, 17], indicating increased utilization of carbohydrates as energy substrate in MLC/mIGF-1 mice.

Interestingly, we did not find differences in total cholesterol, triglycerides, or fasting glucose (Table 1). Total epididymal fat, epididymal fat corrected for body weight and percentage of body fat determined by carcass analysis also showed no difference between WT and MLC/mIGF-1 mice (Table 1).

**Figure 2:**
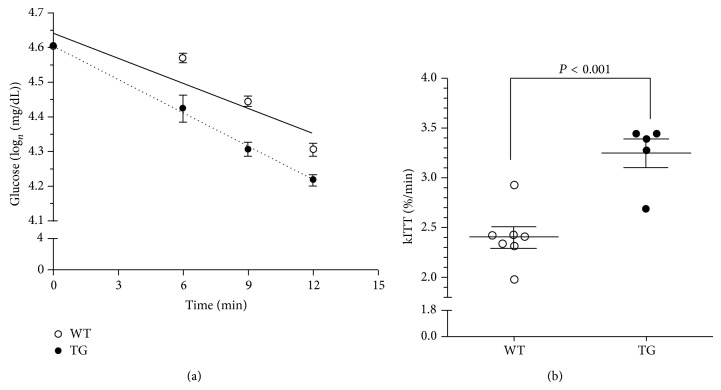
Glucose disposal rate (a) and kITT values (b) for WT (open circles and solid line) and MLC/mIGF-1 animals (filled circles and dotted line). Data expressed as mean ± SEM. *n* = 5–7; ^*^ *P* < 0.001 versus WT.

**Figure 3:**
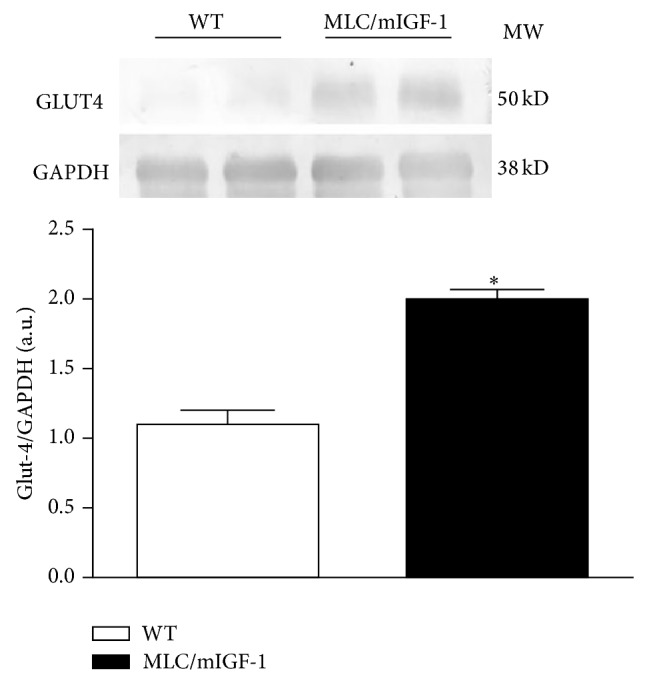
Representative western blot and densitometry of bands for GLUT4 protein expression in EDL muscle from WT (open bars) and MLC/mIGF-1 (filled bars) mice. Histogram represents the ratio of GLUT4:GAPDH densitometry values. Data are expressed as mean ± SEM. *n* = 4; ^*^ *P* < 0.05 versus WT.

Since the main metabolic difference observed between WT and MLC/mIGF-1 animals was a preference for carbohydrates as energy substrate, we determined insulin sensitivity in the animals by performing an ivITT. This test revealed a more pronounced curve of decay in MLC/mIGF-1 mice (Figure 2(a)). kITT determination from the ivITT showed a 2.4%/min and 3.25%/min rate of glucose disposal in WT and MLC/mIGF-1 animals, respectively, which represents a 35% increase in insulin-dependent glucose disposal in MLC/mIGF-1 mice (Figure 2(b)). The higher sensitivity to insulin in these animals was corroborated by higher levels of GLUT4 protein in EDL muscle (Figure 3), with ~2-fold upregulation in this tissue (*P* < 0.05) in comparison with WT groups.

**Figure 4:**
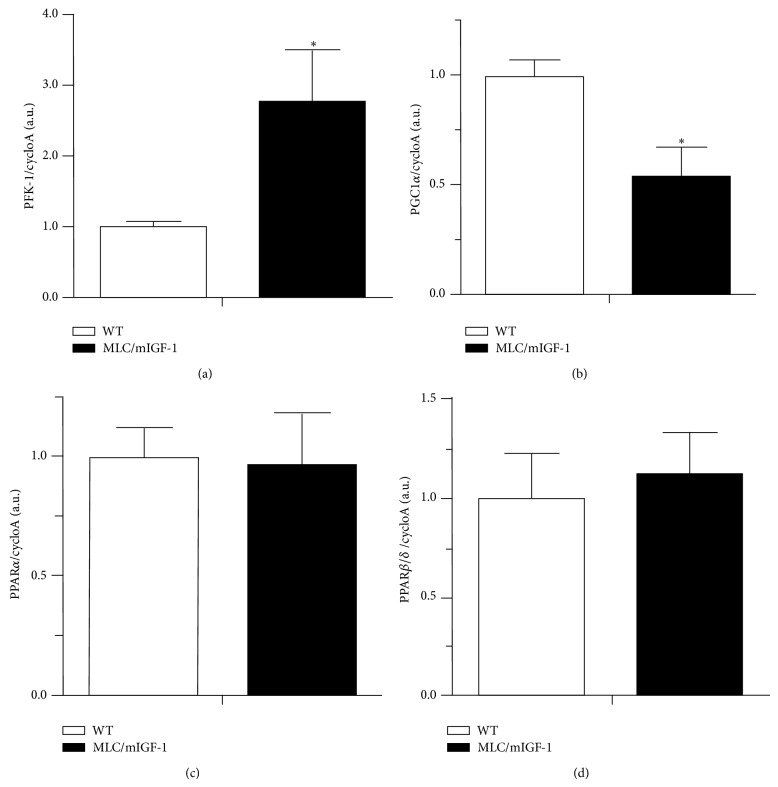
mRNA levels for (a) PFK-1, (b) PGC1*α*, (c) PPAR*α*, and (d) PPAR*β*/*δ* in WT (open bars) and MLC/mIGF-1 (filled bars) mice. CycloA is an internal control. Data are expressed as mean ± SEM. *n* = 3–5; ^*^ *P* < 0.05 versus WT.

Since higher preference for carbohydrates was evident in the whole animal, we decided to investigate whether key genes involved in cell metabolism were affected in the EDL muscle. We found mRNA levels for PFK-1 (Phosphofructokinase 1), a rate-limiting enzyme in the glycolytic pathway [20], to be upregulated ~3-fold in EDL muscle of MLC/mIGF-1 mice compared to WT (Figure 4(a)). We also found mRNA for PGC1*α*, a key regulatory element in mitochondrion biogenesis [21], to be decreased by ~50% in EDL muscle of MLC/mIGF-1 mice compared to WT (Figure 4(b)).

On the other hand, fatty acid metabolism does not appear to be disturbed in EDL muscle of MLC/mIGF-1 mice, since the key regulatory elements, PPAR*β*/*δ* and PPAR*α* [22, 23], were unaltered in these animals in comparison to WT (Figures 4(c) and 4(d)).

## 4. Discussion

In the present study we have used a genetic model in which IGF1 is specifically expressed in fiber type II [8], allowing a better understanding of the specific hypertrophy of those upon metabolic profile.

We have observed quite unique features in MLC/mIGF-1 animal model, noteworthy, MLC/mIGF-1 animals presented relative lower food consumption despite higher muscle mass. Curiously, they have also a shift towards higher utilization of carbohydrate use as a fuel source, which is accompanied by increased insulin sensitivity. Although we have not specifically addressed the involved mechanisms, we reason that the higher whole body insulin sensitivity might be due to higher glucose uptake in type II skeletal muscle fibers, where the IGF-1 transgene is expressed. Indeed, this selective increased sensitivity was correlated with higher levels of GLUT4 protein in EDL suggesting that fast twitch muscles in IGF-1 transgenic animals have more capacity to uptake glucose. It has been described that mIGF-1 overexpression leads to increased activation of the PI3K/Akt/mTOR cascade [24, 25]. Since this cascade is involved in the regulation of GLUT4 expression, it could be a mechanism linking mIGF-1 and GLUT4 overexpression. Future studies are needed to better understand how GLUT4 levels are so highly elevated in MLC/mIGF-1 animals.

A main outcome of the present study was the surprising finding that although the MLC/mIGF-1 animals clearly have increased muscle mass, no proportional increase in food intake was observed. In fact these animals consume, in absolute numbers, similar amount of food. Intriguingly, these animals have higher insulin sensitivity and higher carbohydrate utilization without requiring additional food intake or altered blood total cholesterol or triglycerides. Currently it is not clear why and how MLC/mIGF-1 animals can build more lean mass without more food intake. Possibilities include more efficient substrate gut absorption and less energy expenditure. A recently described myokine called irisin has been associated with activation of brown fat and consequently more energy expenditure in form of heat [26]. In addition, it has been shown that irisin expression is increased by PGC-1 alpha. Interestingly our results show that PGC-1 alpha expression is strongly reduced in MLC/mIGF-1 animals as compared to WT, raising the possibility that decreased activity of the PGC-1-irisin axis might be responsible for sparing the necessary fuel to build increased skeletal muscle in MLC/mIGF-1 animals.

It is currently not clear if the effects of IGF-1 increasing the use of carbohydrates observed in the present study are direct or rather a consequence of secondary changes in type II skeletal muscle fibers. It has been shown that exogenous administration of IGF1 to type 2 diabetes patients can improve insulin sensitivity [27]; therefore, a direct effect is possible. Another nonexclusive possibility involves secondary effects such as type II skeletal muscle fiber hypertrophy. In fact the MLC/mIGF1 animals have a consistent hypertrophy in those fibers and it has been shown that increased muscle mass can improve insulin sensitivity [28]. The metabolic consequence of increased insulin sensitivity in the MLC/mIGF1 animals could include resistance to the establishment of type II diabetes. Future studies submitting mIGF-1 hyperexpression animals to a long-term hypercaloric diet leading to increased body weight and also crossing these animals with ob/ob mice are warranted.

To our knowledge no studies systematically have addressed the effects of IGF1 upon fiber type shift. In our model we found a consistent shift in EDL towards a glycolytic profile. This effect might be mediated by decreasing the levels of PGC-1. It is well described that PGC-1 is a strong fiber type I inducer [29]. Another fiber type I inducer, the calciuneurin-NFATc1 axis, might be modulated by IGF1 increased levels [30]. Accordingly modulation of myogenin levels also remain as a possible mechanism since it has been shown to shift enzyme activity from glycolytic to oxidative metabolism [31].

Although in the present study we have, as expected, detected hypertrophy of type II fibers (IIa/IId and IIb) in EDL muscle, intriguingly we have also seen a decrease in cross-sectional area in fibers IIa in soleus muscle. This unexpected finding might reveal a conditional hypertrophic effect of IGF1. For instance one might envisage that the fiber type I, which is the predominant fiber in this muscle, secretes a factor that can interfere with IGF1 hypertrophic action.

In conclusion, MLC/mIGF-1 mice have improved glucose homeostasis due to GLUT4-mediated higher insulin sensitivity. This improvement leads to a greater rate of carbohydrate utilization by the increased muscle, without interfering with other metabolic parameters.

## Figures and Tables

**Table 1:** Biometric and metabolic parameters of wild type and MLC/mIGF-1 mice.

	Wild type			MLC/mIGF-1			*t*-test
	Mean	SEM	*n*	Mean	SEM	*n*	
Body weight (g)	31.8	0.6	9	37.4	0.3	6	*P* < 0.0001
Animal length	10.2	0.05	9	10.4	0.06	6	n.s.
Daily food intake/body weight	0.21	0.007	6	0.18	0.005	6	*P* < 0.05
Cholesterol (mg/dL)	163	1.4	6	166	0.4	6	n.s.
Triglycerides (mg/dL)	118	3.2	6	115	5.3	5	n.s.
Glucose (mg/dL)	138	9.7	6	150	8.3	6	n.s.
Epididymal WAT (g)	0.4	0.06	9	0.5	0.05	6	n.s.
Epididymal WAT/body weight	1.2	0.2	9	1.3	0.1	6	n.s.
% body fat	4.7	0.6	5	4.0	0.6	3	n.s.
O_2_ consumption (mL/min/kg)	77.7	6.3	7	67.2	4.5	7	n.s.
Respiratory quotient (RQ)	0.74	0.02	7	0.90	0.03	7	*P* < 0.001
